# Cooperative effect of chidamide and chemotherapeutic drugs induce apoptosis by DNA damage accumulation and repair defects in acute myeloid leukemia stem and progenitor cells

**DOI:** 10.1186/s13148-017-0377-8

**Published:** 2017-08-14

**Authors:** Yin Li, Yan Wang, Yong Zhou, Jie Li, Kai Chen, Leisi Zhang, Manman Deng, Suqi Deng, Peng Li, Bing Xu

**Affiliations:** 1 Department of Hematology, Nanfang Hospital, Southern Medical University, Guangzhou, 510515 People’s Republic of China; 2 Department of Hematology, The First Affiliated Hospital of Xiamen University, Xiamen, 361003 People’s Republic of China; 3 Guangzhou Institutes of Biomedicine and Health, Chinese Academy of Sciences, Guangzhou, 510530 People’s Republic of China

**Keywords:** Chidamide, Drug resistance, DNA damage, Acute myeloid leukemia, Leukemia stem and progenitor cells

## Abstract

**Background:**

Many conventional chemotherapeutic drugs are known to be involved in DNA damage, thus ultimately leading to apoptosis of leukemic cells. However, they fail to completely eliminate leukemia stem cells (LSCs) due to their higher DNA repair capacity of cancer stem cells than that of bulk cancer cells, which becomes the root of drug resistance and leukemia recurrence. A new strategy to eliminate LSCs in acute myeloid leukemia (AML) is therefore urgently needed.

**Results:**

We report that a low-dose chidamide, a novel orally active benzamide-type histone deacetylase (HDAC) inhibitor, which selectively targets HDACs 1, 2, 3, and 10, could enhance the cytotoxicity of DNA-damaging agents (daunorubicin, idarubicin, and cytarabine) in CD34^+^CD38^−^ KG1α cells, CD34^+^CD38^−^ Kasumi cells, and primary refractory or relapsed AML CD34^+^ cells, reflected by the inhibition of cell proliferation, induction of apoptosis, and increase of cell cycle arrest in vitro. Mechanistically, these events were associated with DNA damage accumulation and repair defects. Co-treatment with chidamide and the DNA-damaging agent IDA gave rise to the production of γH2A.X and inhibited posttranslationally but not transcriptionally the repair gene of ATM, BRCA1, and checkpoint kinase 1 (CHK1) and 2 (CHK2) phosphorylation. Finally, the combination of chidamide and IDA initiated caspase-3 and PARP cleavage, but not caspase-8 and caspase-9, and ultimately induced CD34^+^CD38^−^ KG1α cell apoptosis. Further analysis of AML patients’ clinical characteristics revealed that the ex vivo efficacy of chidamide in combination with IDA in primary CD34^+^ samples was significantly correlated to peripheral blood WBC counts at diagnosis, while LDH levels and karyotype status had no effect, indicating that the combination regimen of chidamide and IDA could rapidly diminish tumor burden in patients with R/R AML.

**Conclusions:**

These findings provide preclinical evidence for low-dose chidamide in combination with chemotherapeutic agents in treating recurrent/resistant AML as an alternative salvage regimen, especially those possessing stem and progenitor cells.

**Electronic supplementary material:**

The online version of this article (doi:10.1186/s13148-017-0377-8) contains supplementary material, which is available to authorized users.

## Background

Poor long-term clinical outcomes from conventional chemotherapy continue to be a major problem for patients with acute myeloid leukemia (AML), even though significant progress has been achieved in recent years. Leukemia stem cells (LSCs) are only a rare population in leukemia cells, which are presumably responsible for relapse and refractory of AML due to their ability for self-renewal and unlimited repopulating potential. Therefore, the pace of developing a novel anti-leukemic drug targeting LSCs is urgently needed.

Histone deacetylases (HDACs), a family of enzymes participating in the remodeling of chromatin, have a critical role in the epigenetic regulation of gene expression. HDAC inhibitors (HDACis) are considered to possess therapeutic property in various types of cancers, including lung cancer, prostate cancer, breast cancer, multiple myeloma, and lymphoma [1–4]. Besides, many HDACis have been served as a monotherapy drug for cancers’ treatment in clinical trials [3, 4]. Recently, HDAC inhibitors have also emerged as a potent and promising strategy for the treatment of leukemia via inducing differentiation and apoptosis in tumor cells [5].

Conventional chemotherapeutic drugs, such as daunorubicin (DNR), idarubicin (IDA), and cytarabine (Ara-C), remain to be the most preferred and efficacious standard agents for majority of the subtypes of leukemia, even though they fail to completely eliminate LSCs which have become the root of tumor recurrence. The precise anticancer mechanism of DNR/IDA/Ara-C is still not well understood, but it is believed that these agents are involved in DNA damage generation and responses, ultimately leading to mitochondrial dysfunction and apoptosis. Meanwhile, HDAC inhibitors directly involve in chromatin modification by inducing core histone hyperacetylation, resulting in an open chromatin configuration. This relaxation of the chromatin structure would be expected to increase chromatin accessibility, not only facilitating transcription factor binding but also DNA-targeting chemotherapeutic agent [6]. The relevant mechanism mentioned above makes it possible for the use of combination of HDAC inhibitors and conventional chemotherapy.

Chidamide (CS055/Epidaza) is a novel orally active benzamide-type HDAC inhibitor, which selectively targets HDACs 1, 2, 3, and 10, and has entered clinical trials in the USA and China. Numerous studies reveal that chidamide exerts a well-characterized anticancer property in a wide range of tumors, for example, colon cancer, hepatocellular carcinoma, and lymphoma as well as leukemia [7–9]. Simultaneously, our previous works further confirm the tumor suppressor function of chidamide in CD34^+^ human leukemia stem cells while it spared CD34^+^ normal hematopoietic cells [10]. In addition, chidamide also exhibits a synergetic action in pancreatic cancer and non-small-cell lung cancer when combined with other conventional drugs [11, 12]. However, whether chidamide could synergistically enhance the cytotoxicity of conventional chemotherapeutic drug in LSCs remains unclear.

In the present study, it remained to be tested the effects and defined whether and by what mechanism(s) chidamide in combination with chemotherapeutic agents would be active against acute myeloid leukemia stem and progenitor cells. Here, we reported that low-dose chidamide could enhance the cytotoxicity of DNA-damaging agents (daunorubicin, idarubicin, and cytarabine), as well as induce apoptosis and cell cycle arrest in leukemia stem-like cells and primary refractory or relapsed CD34^+^ AML cells, particularly for those carrying high peripheral blood WBC counts (>30 × 10^9^/l) at diagnosis, in association with DNA damage reinforcement or impaired DNA damage repair, and finally initiate caspase-3 and poly-(ADP-ribose) polymerase (PARP) cleavage. Thus, our studies provided a theoretical basis to facilitate the development of a novel combinatorial approach to treat patients with AML.

## Methods

### Reagents

Chidamide (CS055, purity >95%) was supplied by Chipscreen Bioscience Ltd. (Shenzhen, China) and dissolved in dimethyl sulfoxide (DMSO) (Invitrogen, USA) to obtain a stock solution of 50 mM. Idarubicin (IDA), daunorubicin (DNR), and cytarabine (Ara-C) were purchased from Sigma-Aldrich (St Louis, MO) and dissolved in phosphate-buffered saline (PBS) at the concentrations of 10, 100, and 100 mM, respectively. All the above stock solutions were stored at −20 °C. It was diluted to the required concentrations in subsequent experiments with culture medium.

### Cell culture and sorting

The acute myeloid leukemia cell lines KG1α and Kasumi were kindly provided by Prof. PT Liu (Wellcome Trust Sanger Institute, UK) and were cultured in (Iscove's Modified Dulbecco's Medium) IMDM (HyClone, Thermo Scientific, MA, USA) or RPMI-1640 (HyClone, Thermo Scientific, MA, USA) medium supplemented with 10% fetal bovine serum (FBS, Gibco, Life Technologies, NY, USA) and 1% penicillin/streptomycin, respectively. For cell line-sorting experiments, cells were stained with hCD34-APC (eBioscience, USA) and hCD38-PE (ebioscience, USA) for 30 min at 4 °C and were washed twice with PBS/1% FBS. Then the cells were sorted by flow cytometry (FACS Aria IIU, BD).

### Primary samples

Relapsed or refractory acute myeloid leukemia (R/R AML) cases were defined according to the classification in the NCCN guidelines. Twelve cases of R/R AML bone marrow samples were obtained from the Nanfang Hospital, Southern Medical University. The study is approved by the Nanfang Hospital Ethics Review Board in accordance with the Declaration of Helsinki. Acquisition of bone marrow samples was performed with the informed consent of the patients. Major patient characteristics were summarized in Table 1. Mononuclear cells were isolated by density gradient centrifugation using Lymphoprep™ (Axis-Shield, Norway) and cultured in IMDM (HyClone, USA) supplemented with 10% fetal bovine serum (Natocor, Argentina), 100 U/ml penicillin, and 100 μg/ml streptomycin (1 × P/S).

**Table 1:** The clinical characteristic of AML patients (*n* = 12)

No.	Age/sex	Disease status	FAB subtype	WBC count(×10^9^/l)	Karyotype	Molecular features	LDH
1	48/M	Refractory	M0	4.11	46,XY	EGR1,CBFβ,MYH1,TP53	High
2	48/F	Refractory	M2b	5.0	46,XY	AML1/ETO	Normal
3	18/F	Refractory	M5b	39.79	46,XY	NPM1,TET2,ASXL1	Normal
4	26/F	Relapse	M0	124.7	46,XY	FLT3-ITD	Normal
5	52/F	Refractory	M2	1.54	Complex	MLL,EGFR	Normal
6	25/F	Refractory	M1	6.6	46,XY	CEBPa	High
7	46/F	Relapse	M2	37.85	46,XX,t(8,21)	AML1/ETO	High
8	23/M	Relapse	M0	1.97	46,XX	AML1/ETO	Normal
9	30/M	Refractory	M0	93.13	46,XX,t(8,21)	CBFβ,MYH1	High
10	42/M	Relapse	M2	33.48	Complex	c-kit	High
11	26/M	Refractory	M5b	5.37	Complex	CEBPa, MLL	High
12	54/M	Relapse	M4eo	79.0	Complex	CEBPa,KMT3A	Normal

*M* man, *F* female, *FAB* French-American-Britain, *WBC* white blood cell, *LDH* lactic dehydrogenase

### CCK-8 assay

The cytotoxic effects of Ara-C, DNR, and IDA with or without chidamide on CD34^+^CD38^−^ KG1α or Kasumi cells were determined by Cell Counting Kit-8 (CCK-8, Dojindo, Kumamoto, Japanese) assay. Cells (3 × 10^4^ cells/well) were seeded in 96-well plates containing 100 μl growth medium and treated with designated doses of Ara-C, DNR, or IDA in combination with or without 0.75 μM chidamide and incubated at 37 °C in a 5% CO_2_ incubator for 24, 48, and 72 h; CCK-8 reagents (10 μl/well) were then added and continued to incubate for an additional 2 h; finally, the absorbances were detected at 450 nm by microplate reader (ELx800, BioTek, USA). The data from three independent triplicates were expressed as a percentage of dead cells compared to a control from the same experiment. Statistical analysis and IC50 determination were calculated by SPSS 20.0.

### Annexin V-APC/PI double-staining apoptosis assay

To assess apoptosis, CD34^+^CD38^−^ KG1α or Kasumi cells were cultured as described above for 24, 48, or 72 h with or without chidamide, Ara-C, DNR, or IDA, then double labeled with Annexin V-APC/PI (eBioscience, San Diego, CA, USA) for 15 min at room temperature in the dark according to the manufacturer’s instructions. Primary samples were stained with hCD34-APC (eBioscience, USA) and Annexin V-FITC/PI to assess the apoptosis of CD34^+^ primary cells induced by chidamide or IDA alone or the two drugs in combination. The stained cells were analyzed by flow cytometry (FACS Fortessa, BD Biosciences). Apoptotic cells were defined as Annexin V positive.

### Cell cycle analysis by PI staining and flow cytometry

CD34^+^CD38^−^ KG1α cells were exposed to 0.5 or 0.75 μM chidamide in combination with or without 5 or 10 nM IDA for 72 h, with an untreated group as the control. Cells were harvested, washed with PBS, and fixed in 70% ethanol at 4 °C overnight. The cells were washed with PBS, resuspended in PBS containing 10 μg/ml RNase A and 0.1% Triton X-100, and incubated at 37 °C for 30 min. Subsequently, 50 μg/ml propidium iodide (PI) was added, and the cells were incubated at room temperature in the dark for 30 min. The samples were analyzed for DNA content by flow cytometry (FACS Calibur, BD Biosciences).

### Quantitative analysis of γH2A.X by flow cytometry

CD34^+^CD38^−^ KG1α cells were exposed to 20 or 40 nM IDA in combination with or without 0.75 μM chidamide for 24 h, with an untreated group as the control. Cells were harvested and incubated for 15 min on ice in a hybridization buffer (PBS containing 0.5% bovine serum albumin (BSA) and 0.25% Triton X-100). After centrifugation, the cells were incubated with rabbit monoclonal anti-γH2A.X antibody (Cell Signaling Technology, USA) for 1 h, then washed with PBS and incubated with an FITC-conjugated mouse anti-rabbit IgG antibody (BD Pharmingen) for 30 min in the dark at room temperature. The stained cells were analyzed by flow cytometry (FACS C6, BD Biosciences).

### DNA damage analysis by γH2A.X foci immunofluorescence

CD34^+^CD38^−^ KG1α cells were cultured with or without 40 nM IDA and 0.75 μM chidamide for 24 h. Cells were harvested and dropped in glass coverslips, then were fixed with 4% paraformaldehyde for 20 min, followed by three PBS rinses, permeabilized with 0.1% Triton X-100 (Sigma) for 15 min and blocked with 5% BSA in PBS for 1 h at room temperature (RT). The samples were then stained overnight at 4 °C with primary antibody against *γH2A.X* (1:200, Cell Signaling, Herts, UK), followed by incubation with FITC goat anti-rabbit IgG (Sigma) for 1 h at RT in the dark, and then were counterstained using DAPI. Subsequently, the coverslips were mounted on glass slides and cell nuclei. The cells were scanned and images were captured by confocal fluorescence microscope.

### Total RNA isolation and qRT-PCR

Total RNA was isolated using TRIzol reagent (Invitrogen, Paisley, UK) according to the manufacturer’s protocols. RNA (1 μg) was reverse transcribed into cDNA using RT reagent kit (TaKaRa, Dalian, China). The quantitative real-time polymerase chain reactions were performed using TransStart Tip Green qPCR Supermix (Transgene, China) and were run on the CFX96 (Bio-Rad, USA) following the instruction of the supplier. The human housekeeping gene β-actin (XR018317) was used as the RNA-loading control. Additional file 1 shows the sequences of the primers and the sizes of the amplified fragments. The RT-PCR conditions were as follows: 1 cycle at 94 °C for 10 min; 40 cycles at 94 °C for 10 s, 60 °C for 30 s; and 1 cycle at 72 °C for 3 min.

### Western blotting analysis

CD34^+^CD38^−^ KG1α cells (5 × 10^5^/ml) were cultured for 24 or 48 h in the absence or presence of 40 nM IDA and 0.75 μM chidamide. The protein expression levels were determined by staining with primary antibodies and relevant HRP-conjugated secondary (1:10,000, Abcam, Cambridge, UK) antibodies. The primary antibodies (caspase-3, caspase-8, caspase-9, and PARP—Beyotime, China; p-BRCA1, p-ATM, p-CHK1, p-CHK2, γH2A.X, and Ace-H3—Cell Signaling, Herts, UK) were diluted at 1:1000 in 5% fat-free milk-TBST. Anti-β-actin (1:1000, Cell Signaling, Herts, UK) was used as a loading control. The signal was detected using an ECL Western Blotting Detection Kit (GeneFlow, Staffordshire, UK).

### Statistical analysis

Data were expressed as the mean ± standard deviation (S.D.) of at least three independent experiments. Statistical analyses were performed by Student’s *t* test or one-way analysis of variance (ANOVA) using SPSS 20.0 software. *P* ≤ 0.05 was regarded as statistically significant.

## Results

### Low-dose chidamide enhanced cytotoxicity of chemotherapy agents in leukemia stem-like cells

**Fig. 1:**
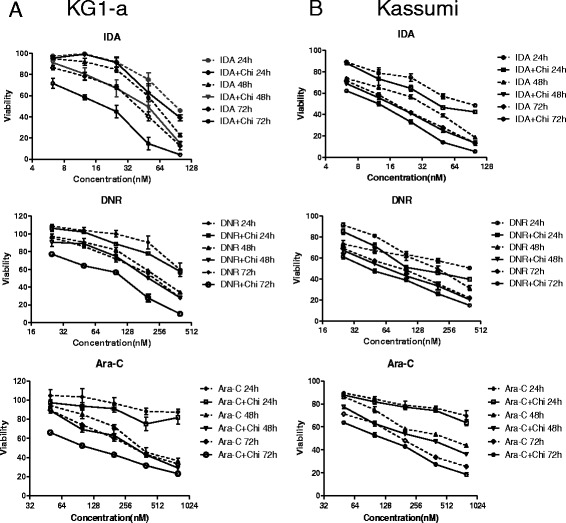
Chidamide enhanced IDA, DNR, or Ara-C in cytotoxic effects on CD34^+^CD38^−^ KG1α and Kasumi cells. CD34^+^CD38^−^ KG1α (**a**) and Kasumi (**b**) cells were exposed to the indicated concentrations of IDA, DNR, or Ara-C with or without 0.75 μM chidamide for 24, 48, and 72 h, after which the cell viability effect was analyzed by CCK-8 assay. Percent of viability is normalized with DMSO-treated control. The values in the figure are expressed as the mean ± S.D. from three independent experiments

To explore whether low-dose chidamide might influence the cytotoxicity of chemotherapy agents in leukemia stem cell-like cells (LSC-like cells), we investigated the anti-proliferative activities of IDA, DNR, or Ara-C alone or in combination with 0.75 μM chidamide in CD34^+^CD38^−^ KG1α cells by CCK-8 assay at 24-, 48-, and 72-h treatments. Cell viability curves were shown in Fig. 1a. Either IDA, DNR, or Ara-C alone or in combination with chidamide inhibited the proliferation of CD34^+^CD38^−^ KG1α cells in a dose- and time-dependent manner. The IC50 values were shown in Table 2. The IC50 values of IDA, DNR, or Ara-C coupled with chidamide were less than single-agent treatment, and *P* values were less than 0.05 after 48- and 72-h exposures, while there was no significance for the 24-h treatment (*P* > 0.05). Analogous results were obtained in another leukemia stem-like cell line, CD34^+^CD38^−^ Kasumi cells. Notably, the *P* values were all less than 0.05 except treatment with chidamide plus Ara-C for 24 h (Fig. 1b and Table 2). Taken together, these results indicated that whereas chemotherapy agents themselves were active against acute myeloid leukemia stem-like cell lines, combined administration with non-toxic concentrations of chidamide (e.g., 0.75 μM) remarkably potentiates the cytotoxicity of chemotherapy agents, primarily via inhibition of cell proliferation in a dose- and time-dependent manner.

**Table 2:** Chidamide enhanced the cytotoxicity of IDA, DNR, or Ara-C to CD34^+^CD38^−^ KG1α and Kasumi cells in vitro

Drug	IC50 (nM) of KG1α		Fold	*P*	IC50 (nM) of Kasumi		Fold	*P*
	Single	Combination			Single	Combination		
IDA								
24 h	92.36 ± 7.26	75.65 ± 14.36	1.22	0.146	85.67 ± 11.61	53.86 ± 10.75	1.59	0.025
48 h	56.96 ± 5.64	39.17 ± 6.50	1.45	0.023	26.13 ± 2.53	15.63 ± 1.21	1.84	0.003
72 h	40.99 ± 15.57	17.23 ± 5.88	2.38	0.024	17.23 ± 1.54	11.33 ± 0.95	1.52	0.005
DNR								
24 h	500 ± 17.58	468.56 ± 21.35	1.07	0.209	324.81 ± 34.75	171.71 ± 32.41	1.89	0.005
48 h	257.90 ± 9.66	205.82 ± 7.23	1.25	0.002	160.97 ± 21.54	67.95 ± 8.09	2.37	0.002
72 h	224.20 ± 5.80	95.78 ± 5.91	2.34	<0.001	80.52 ± 13.36	47.20 ± 5.57	1.71	0.016
Ara-C								
24 h	>10^6^	>10^6^	–	–	7920.75 ± 5131.77	3842.19 ± 2732.90	2.06	0.291
48 h	435.82 ± 51.23	313.13 ± 48.44	1.39	0.039	476.32 ± 29.21	300.70 ± 34.38	1.58	0.003
72 h	334.27 ± 28.76	127.84 ± 11.21	2.61	<0.001	181.46 ± 10.77	115.85 ± 6.83	1.54	0.001

Note: CD34^+^CD38^−^ KG1α and Kasumi cells were exposed to IDA, DNR, or Ara-C with or without chidamide (0.75 μM) for 24, 48, and 72 h, with cytotoxicity being assessed using a CCK-8 assay

### Chidamide synergized IDA-, DNR- or Ara-C-induced apoptosis in both leukemia stem-like cells and primary relapsed or refractory AML CD34^+^ cells

**Fig. 2:**
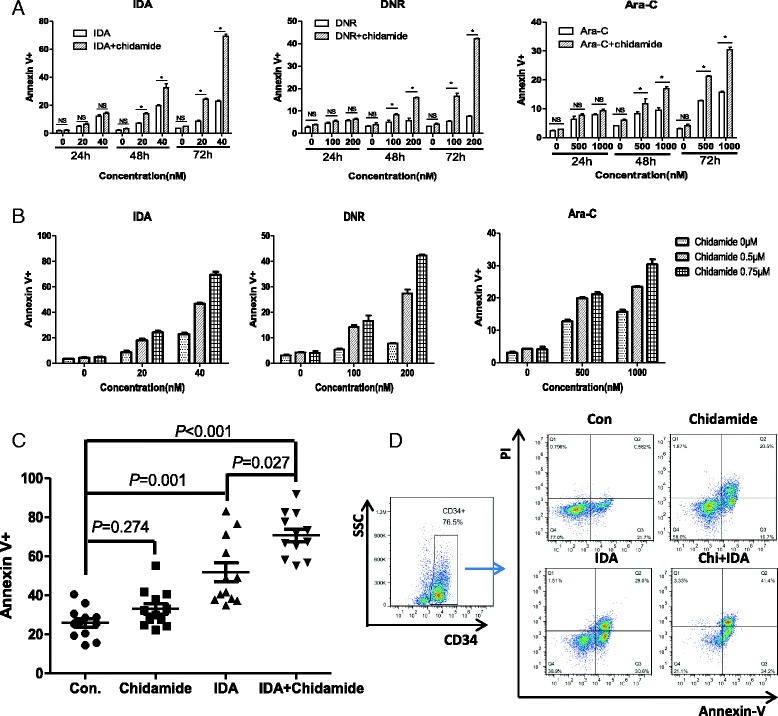
Chidamide synergized IDA-, DNR-, or Ara-C-induced apoptosis in both CD34^+^CD38^−^ KG1α cells and primary relapsed or refractory AML CD34^+^ cells. CD34^+^CD38^−^ KG1α cells were exposed to the indicated concentrations of IDA, DNR, or Ara-C with or without 0.75 μM chidamide for 24, 48, and 72 h (**a**), with or without 0.5 or 0.75 μM chidamide for 72 h (**b**), after which flow cytometric analysis was performed to determine the percentage of Annexin V^+^ cells. *Horizontal lines* represent the mean ± S.D. from three independent experiments. (**c**) Primary CD34^+^ AML cells were exposed to the 20 nM IDA with or without 0.75 μM chidamide, after which apoptotic ratios were determined by Annexin V staining and flow cytometry. (**d**) Representative data for flow cytometric analysis of hCD34 and Annexin V/PI staining in primary cells after exposed (48 h) to 20 nM IDA with or without 0.75 μM chidamide. *P < 0.05

Efforts were then undertaken to determine the effects of low-dose chidamide on IDA-, DNR-, or Ara-C-induced apoptosis by treating CD34^+^CD38^−^ KG1α cells for 24, 48, and 72 h. The apoptotic cells were assessed by Annexin V-APC/PI double labeling and flow cytometric analysis. There was no significant difference between the control and the single low-dose chidamide (0.75 μM)-treated groups nor difference between chidamide in combination with different concentrations of IDA and the single-agent treatment groups after 24 h treatment(*P* > 0.05). Treatment with IDA alone resulted in increase in apoptosis, especially after the 72-h treatment; nevertheless, different concentrations of IDA in combination with chidamide significantly induced apoptosis in a concentration-dependent manner compared with IDA or chidamide alone for the 48- and 72-h treatments (*P* < 0.05, Fig. 2a). Similar results were observed for DNR or cytarabine alone as well as in combination with chidamide (Fig. 2a).

Since the killing effect of combination treatment was improved at 72 h, we further evaluated the combined effect by testing different concentrations of chidamide with or without different concentrations of chemotherapy agents for 72 h. The combination index (CI) values were calculated according to the Chou-Talalay equation (Fig. 2b, Table 3). The CI values for the combination consistently indicated significant synergy, as defined by CI <1. The mean CIs were all less than 1 in IDA, DNR, or Ara-C in combination with chidamide, respectively, suggesting a good synergy. The analogical results were presented in Kasumi cells (data not show; see Additional files 2 and 3: Figure S1 and Table S2).

**Table 3:** Summary of CIs generated from the isobologram of increasing concentrations of chidamide and IDA, DNR, or Ara-C for CD34^+^CD38^−^ KG1α cells

Drugs (nM)	FA	Chidamide (μM)	FA_Chidamide_	FA_Comb_	CI
IDA	FA_IDA_				
20	0.087	0.5	0.044	0.179	0.610
20	0.087	0.75	0.049	0.242	0.481
40	0.229	0.5	0.044	0.468	0.516
40	0.229	0.75	0.049	0.693	0.291
DNR	FA_DNR_				
100	0.053	0.5	0.041	0.142	0.149
100	0.053	0.75	0.042	0.166	0.107
200	0.077	0.5	0.041	0.274	0.069
200	0.077	0.75	0.042	0.422	0.022
Ara-C	FA_Ara-C_				
500	0.128	0.5	0.041	0.200	0.220
500	0.128	0.75	0.042	0.212	0.177
1000	0.157	0.5	0.041	0.235	0.244
1000	0.157	0.75	0.042	0.304	0.089

CI less than 1.0 indicates synergistic effect. FA_Chidamide_ indicates fraction of cytotoxicity by chidamide alone; FA_IDA_, FA_DNR_, and FA_Ara-C_, fraction of cytotoxicity by IDA, DNR, or Ara-C alone, respectively; FA_Comb_ fraction of cytotoxicity by chidamide plus IDA, DNR, or Ara-C

We then tested the activity of chidamide in combination with IDA in CD34^+^ primary patient samples (bone marrow mononuclear cells) obtained from adult refractory or relapsed AML. The clinical characteristics of these AML patients are summarized in Table 1. Consistent with the anti-leukemic activity of chidamide plus IDA observed in the CD34^+^CD38^−^ KG1α cell line, co-treatment (48 h) with 0.75 μM chidamide and 20 nM IDA resulted in significant increases in apoptosis of primary CD34^+^ R/R AML cells (*P* < 0.05 vs untreated control, *n* = 12; Fig. 2c, d), although the responses varied among patients. Further analysis on the AML patients’ clinical characteristics revealed that the ex vivo efficacy of chidamide in combination with IDA in primary CD34^+^ samples was significantly correlated to peripheral blood WBC counts at diagnosis (*P* = 0.016). However, the status of refractory or relapse, LDH level, and karyotype did not significantly affect response of primary CD34^+^ AML cells to chidamide in combination with IDA (Table 4), indicating that the combination regimen of chidamide and IDA could rapidly diminish tumor burden in a patient with R/R AML. Thus, chidamide synergistically enhanced chemotherapy agent-induced cell apoptosis in leukemia stem-like cells and primary refractory or relapsed AML CD34^+^ cells.

**Table 4:** The relationship between clinical characteristics and cytotoxicity of the regimens combining chidamide with IDA in primary refractory or relapsed CD34^+^ AML cells

Characteristic	Group	Apoptotic cells (%)				
		Con.	Chidamide	IDA	Chi + IDA	*P* value
Status	Refractory (*n* = 7)	25.86 ± 8.36	34.30 ± 11.07	53.84 ± 18.84	70.00 ± 13.96	0.732
	Relapse (*n* = 5)	26.20 ± 7.74	31.52 ± 7.47	49.21 ± 15.93	72.00 ± 7.64	
WBC	>30 (*n* = 6)	27.54 ± 9.79	36.05 ± 11.21	58.28 ± 18.96	76.63 ± 9.60	0.016*
(×10^9^/l)	<30 (*n* = 6)	24.46 ± 5.50	30.24 ± 7.10	45.54 ± 13.60	65.00 ± 10.46	
Karyotype	Non-complex (*n* = 8)	25.36 ± 9.57	31.61 ± 10.84	49.45 ± 16.28	68.62 ± 11.37	0.178
	Complex (*n* = 4)	27.30 ± 1.64	36.19 ± 5.89	56.83 ± 20.07	75.17 ± 11.50	
LDH	Normal (*n* = 6)	24.77 ± 6.94	30.88 ± 7.39	50.48 ± 16.02	66.97 ± 9.98	0.221
	High (*n* = 6)	27.24 ± 8.94	35.41 ± 11.37	53.34 ± 19.49	74.65 ± 12.16	
Total		26.00 ± 7.74	33.14 ± 9.45	51.91 ± 17.08	70.81 ± 11.34	<0.01

*WBC* white blood cell, *LDH* lactic dehydrogenase, *IDA* idarubicin, *Con*. control group, *Chi* chidamide

### Chidamide potentiated IDA-induced cell cycle arrest in CD34^+^CD38^−^ KG1α cells

**Fig. 3:**
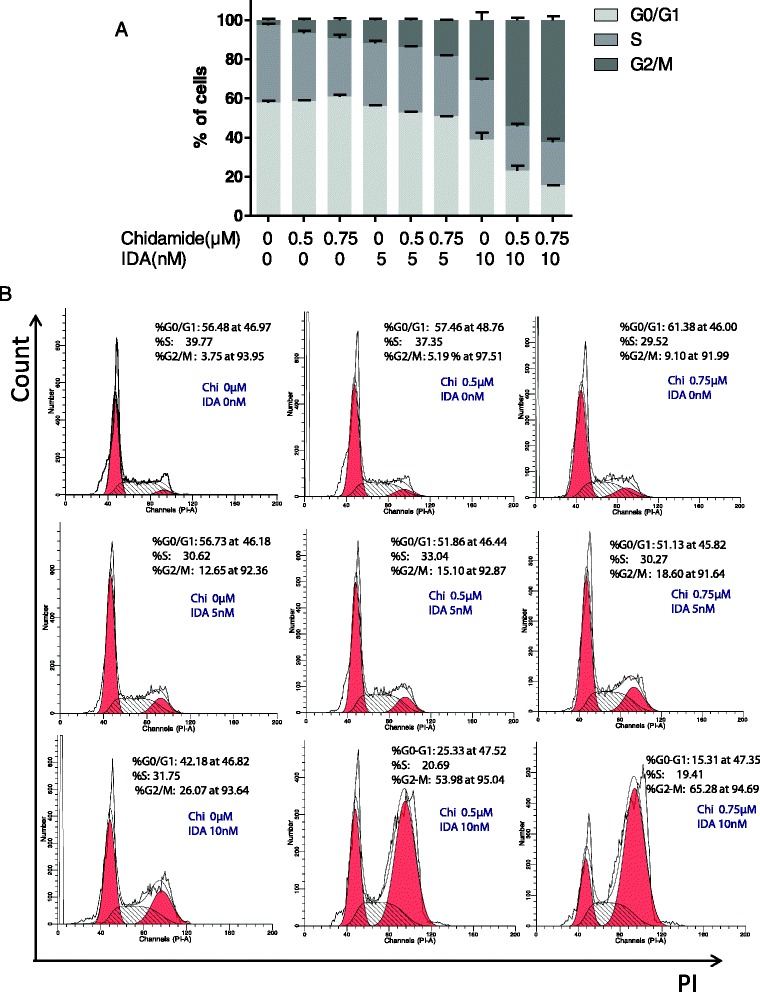
Chidamide intensified IDA-induced cell cycle arrest in CD34^+^CD38^−^ KG1α cells. (**a**) CD34^+^CD38^−^ KG1α cells were exposed to 5 or 10 nM IDA with or without 0.5 or 0.75 μM chidamide for 72 h, after which flow cytometric analysis was performed to determine the cell cycle. *Horizontal lines* represented the mean ± S.D. from three independent experiments. (**b**) Representative data for flow cytometric analysis of PI staining in CD34^+^CD38^−^ KG1α cells after exposed (72 h) to 5 or 10 nM IDA with or without 0.5 or 0.75 μM chidamide

To further characterize the role of chidamide in combination with IDA, we subsequently performed FACS analysis of propidium iodide-stained cells treated with two drugs alone or in combination to investigate their impact on the cell cycle. It appeared that a certain fraction of the combination group retained in the G2/M-phase, which was significantly higher than the single-drug group. Simultaneously, FACS analysis revealed that fewer cells of the combination treatment group were in the S-phase as compared to untreated and 10 nM IDA controls, while there was no significant difference compared to the 5 nM IDA controls. The percentage of cells detected in the G0/G1-phase did not differ significantly between the combination treatment group and untreated or 5 nM IDA controls, while fewer cells of the combination treatment group were in the G0/G1-phase as compared to 10 nM IDA controls (Fig. 3).

### Chidamide increased IDA-induced DNA damage in CD34^+^CD38^−^ KG1α cells

**Fig. 4:**
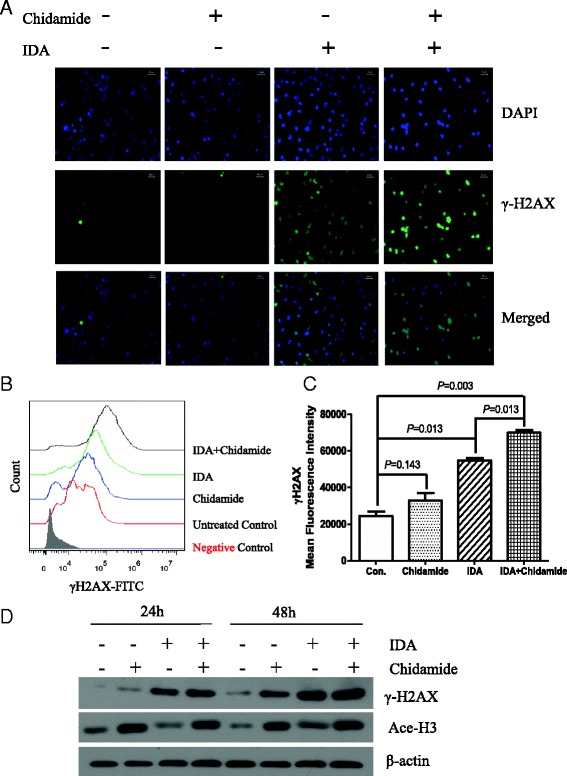
Chidamide increased IDA-induced DNA damage in CD34^+^CD38^−^ KG1α cells. CD34^+^CD38^−^ KG1α cells were treated with or without 0.75 μM of chidamide in combination with 40 nM IDA for 24 h. (**a**) Immunostaining was performed with γH2A.X and counterstained with DAPI. Each experiment was performed in triplicate. (**b**) The expression of γH2A.X was examined in the presence or absence of chidamide with or without IDA for 24 h followed by flow cytometry. *Filled-area histograms* represent medium treated cells, whereas *color histograms* represent different treated group, and the compiled results from three experiments are shown in (**c**). (**d**) The expression of γH2A.X and acetylation of histone 3 were examined in the presence or absence of chidamide with or without IDA for 24 and 48 h followed by western blot

DNA damage was considered as one of the most important molecular effects and cell death mechanisms induced by chemotherapy agents. Levels of phosphorylated H2A.X on Ser139 (γH2A.X), a marker of DNA (DNA doubled-strand breaks) DSBs, were measured by immunofluorescence confocal microscopy following IDA, chidamide, and dual drug treatment. As shown in Fig. 4a, DMSO-treated CD34^+^CD38^−^ KG1α cells (negative control) had quite low background levels of γH2A.X, and there was no significant difference between the 0.75 μM chidamide-treated and negative control groups, while levels of phosphorylated H2A.X were sharply higher than those in the control or chidamide-alone group after treating with 40 nM IDA. This indicated that the 0.75 μM chidamide did not induce distinct DNA damage while the 40 nM IDA or the combination of IDA and chidamide could make quite a lot DNA strand breaks in CD34^+^CD38^−^ KG1α cells; besides, cells exposed to chidamide and IDA simultaneously increased the expression of γH2A.X much more than the IDA alone.

The above results were further supported by the assessment of phosphorylation of histone H2A.X using flow cytometry in CD34^+^CD38^−^ KG1α cells after 40 nM IDA, 0.75 μM chidamide, or their combination. As showed in Fig. 4b, c, the phosphorylation of histone H2A.X was not altered after being treated with chidamide alone compared with the negative group. Higher mean fluorescence intensity (MFI) and more γH2A.X-positive cells were consistently observed at 24 h after chidamide was incorporated in the combination group than in IDA alone. The levels of γH2A.X were significantly increased in IDA alone and in combination with the chidamide group compared to negative counterparts. Meanwhile, the expression of γH2A.X appeared higher in the combination group compared to IDA alone. Similar results were confirmed by western blot analysis (Fig. 4d).

To further confirm that the above observed effects to reflect chidamide’s function in inhibiting HDACs, we assessed acetylation of histone H3. Histone H3 was highly acetylated (see Fig. 4d).

### Chidamide posttranslationally but not transcriptionally inhibited the repair of IDA-induced DNA damage in CD34^+^CD38^−^ KG1α cells

**Fig. 5:**
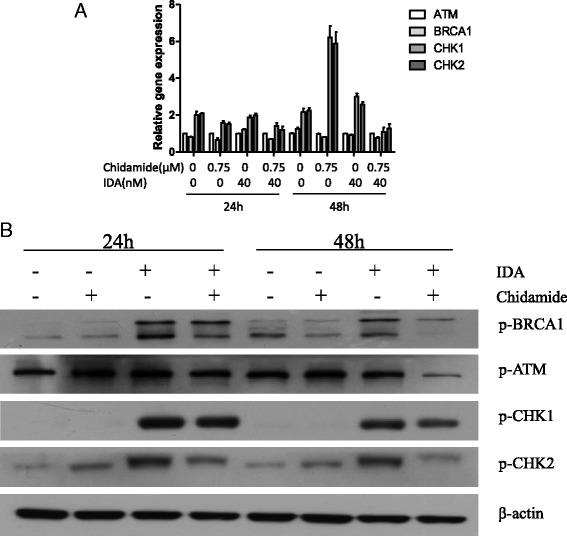
Chidamide posttranslationally but not transcriptionally inhibited the repair of IDA-induced DNA damage in CD34^+^CD38^−^ KG1α cells. CD34^+^CD38^−^ KG1α cells were incubated with 0.75 μM chidamide ±40 nM IDA for 24 and 48 h, followed by qRT-PCR to evaluate the gene expression of BRCA1, ATM, CHK1, and CHK2 (**a**), or western blot analysis to monitor the expression of p-BRCA1, p-ATM, p-CHK1, and p-CHK2 (**b**). β-actin was used as a loading control

To assess whether DNA damage repair was implicated in the increasing apoptosis, we first evaluated the genes involved in DNA damage repair via qRT-PCR analysis. CD34^+^CD38^−^ KG1α cells were cultured with chidamide (0.75 μM), IDA (40 nM), or the combination for 24 and 48 h. As shown in Fig. 5a, the gene expression of BRCA1, ATM, CHK1, and CHK2 were significantly increased in IDA alone or in combination with the chidamide group as compared to untreated and 0.75 μM chidamide controls, However, the gene expression of BRCA1, ATM, CHK1, and CHK2 did not differ significantly between the combination and IDA alone. Then we test the phosphorylated protein of BRCA1, ATM, CHK1, and CHK2 by western blot analysis. The results showed that p-BRCA1, p-ATM, p-CHK1, and p-CHK2 were induced by IDA alone or in combination with chidamide; however, the co-treatment with chidamide and IDA induced a significantly decreased expression of p-BRCA1, p-ATM, p-CHK1, and p-CHK2 than the IDA alone, indicating that chidamide can posttranslationally but not transcriptionally inhibit the IDA-induced expression of DNA damage repair gene (Fig. 5b).

### Chidamide in combination with IDA initiated caspase-3 and PARP cleavage in CD34^+^CD38^−^ KG1α cells

**Fig. 6:**
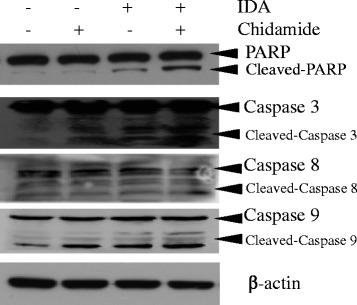
Chidamide initiated caspase-3 and PARP cleavage in CD34^+^CD38^−^ KG1α cells. Western blotting analysis of the expression of caspase-3, caspase-8, caspase-9, and PARP and their cleavage in CD34^+^CD38^−^ KG1α cells exposed to 0.75 μM chidamide with or without 40 nM IDA treatment for 48 h, with the untreated group as the control. β-actin was used as a loading control

To further investigate the molecular mechanism of action for chidamide in combination with IDA treatment to induce CD34^+^CD38^−^ KG1α cell apoptosis after DNA damage, the levels of caspase-3, caspase-8, caspase-9, and poly-(ADP-ribose) polymerase (PARP) were determined after 48 h of treatment by using western blot analysis. As show in Fig. 6, chidamide alone did not induce any noticeable change in cleaved caspase-3 and PARP protein levels; however, IDA alone and in combination with chidamide both markedly induced cleavage of caspase-3 and PARP, with the generation of active, low molecular weight cleaved fragments. Furthermore, a significant increase of these protein levels in IDA plus chidamide compared to IDA-alone treatment was seen. Treatment with chidamide plus IDA and the two compounds alone enhanced caspase-9 cleavage, as compared to untreated control (Fig. 6), but no effect was observed with either of the two drugs used alone or the combination. Neither two drugs alone nor in combination affected the protein levels of caspase 8 (Fig. 6).

## Discussion

Cancer stem cells were responsible for tumor initiation, growth, metastasis, and therapeutic resistance. The objective was to eliminate cancer stem cells to overcome disease resistance while preventing recurrence remained a significant therapeutic obstacle in numerous malignancies. Therefore, novel therapeutic strategies were needed for acute myeloid leukemia stem cells. We had previously shown that selective HDAC inhibitor chidamide can preferentially eliminate primary AML stem and progenitor cells while sparing normal hematopoietic cells [10]. In this preclinical setting, we further demonstrated that a low dose of chidamide can sensitize conventional chemotherapeutics to leukemia stem cell-like cells, including CD34^+^CD38^−^ KG1α cells, CD34^+^CD38^−^ Kasumi cells, and primary AML stem/progenitor cells.

We first evaluated the cytotoxic effect of chidamide in combination with conventional chemotherapy agents in CD34^+^CD38^−^ KG1α and Kasumi cells. The results showed that the anti-proliferative activities were increased in the combination of IDA, DNR, or Ara-C with chidamide compared with single-agent treatment. Consistently, the combination was significantly more effective in inducing apoptosis in leukemia stem-like cells than the single-agent treatment. The CI values for the combination indicated significant synergy as well. FACS analysis revealed that treatment with chidamide plus IDA resulted in accumulation of cells in the G2/M-phase and a reduction of cells in S and/or G0/G1. It was noteworthy that chidamide was not the only HDACi that gave the combinatorial drug phenotypes. SAHA in combination with IDA, DNR, or Ara-C also effectively inhibited CD34^+^CD38^−^ KG1α cell proliferation and induced cell apoptosis in a dose- and time-dependent manner (see Additional file 4: Figure S2).

Importantly, low-dose chidamide also dramatically enhanced lethality of IDA in primary refractory or relapsed AML CD34^+^ cells. Combined treatment with chidamide and IDA interestingly induced higher percentage of apoptosis in blasts of patients with WBC count >30 × 10^9^/l than those with <30 × 10^9^/l, suggesting that this combination regimen might overcome the unfavorable effects of high tumor burden. It was reported recently that somatic TP53 mutation consolidated the development of preleukemic stem cells and poor prognosis in AML [13]. After receiving the combination therapy of chidamide with IDA, the percentage of Annexin V-positive cells was 56.895 ± 3.104 in patient 1 who had TP53 mutation, which was lower than the average of the others (72.072 ± 10.971), thus indicating that TP53 mutation may be likely to affect apoptosis response to combined drug treatment. However, further studies including more cases are needed to confirm these findings.

HDAC inhibitors can kill cancer cells by acetylating histone to modify chromatin structure and gene expression [14]. Thus, we first evaluated the acetylation of histone 3. Figure 4d showed that the levels of H3 acetylation upregulated in both chidamide alone and in combination with IDA. Nevertheless, it could not elucidate the cell death mechanisms being evoked by conventional chemotherapy agents alone or in combination with chidamide which killed much more cells than the control and chidamide groups. Thus, new signaling routes may be affected by the drug combination.

Idarubicin (IDA), daunorubicin (DNR), and cytarabine (Ara-C) were the most effective and frequently used DNA-damaging anticancer drugs for the treatment of patients with AML [15]. This led us to investigate the potential role of DNA damage in the cell death mechanism. The results showed that the levels of phosphorylated H2A.X were significantly increased in IDA alone and in combination with the chidamide group compared to negative counterparts and chidamide alone, and the expression of γH2A.X appeared higher in the combination group compared to IDA alone.

Resistance was a major barrier for successful therapy with conventional chemotherapy agents, leading to a poor clinical outcome. DNA damage was considered as one of the most important molecular effects and cell-killing mechanisms induced by anthracycline drugs; however, DNA-damaging chemotherapy also activates DNA damage response (DDR) mechanisms, which were considered to be one of the major mechanisms of drug resistance for cancer stem cells [16]. Cancer stem cells enhanced DNA repair capacity to process DNA damage more efficiently than bulk cancer cells to maintain genomic integrity while exposed to DNA-damaging agents. It had been reported that CD133^+^ (cancer stem cell) CSCs isolated from the A549 human lung carcinoma cell line were found to enhance repair of DNA DSBs and to upregulate the expression of DSB repair genes [17]. Similarly, DDR and the expression of numerous repair proteins were also found to be highly upregulated in Lin-CD29hCD24h tumor-initiating cells isolated from mouse mammary gland tumors [18]. It also demonstrated that the expression of DNA repair relative genes, such as BRCA1 and rad51, increased significantly in pancreatic putative CSCs compared with bulk cells [19]. CD133^+^ glioma stem cells contribute to glioma radio resistance and tumor regeneration through enhanced cell cycle checkpoint response and DNA repair [20]. Thus, modulation of the DDR in CSCs became a potential therapy strategy in the treatment with DNA damaging.

In order to overcome the resistance and increase DNA damage, some studies had implicated HDAC inhibitors in the transcriptional downregulation of DNA-repairing factors as a potential mechanism for maintaining γH2A.X and sensitivity of cancer cells to DNA-damaging agents [21]. HDAC inhibitors had been shown to upregulate and sustain γH2A.X expression by downregulating the expression of DNA repair proteins in cancer cells, including prostate, lung, melanoma, and AML [22–25]. This prompted us to further detect the change of DNA repair factors. We first evaluated the genes involved in DNA damage repair via qRT-PCR analysis. The gene expression of BRCA1, ATM, CHK1, and CHK2 were significantly increased in IDA alone or in combination with the chidamide group as compared to the untreated and chidamide-alone controls, but there was no significant difference between the combination and IDA alone. Then we tested phosphorylated protein of BRCA1, ATM, CHK1, and CHK2 by western blot analysis. We found that the expression of p-ATM, p-BRCA1, p-CHK1, and p-CHK2 induced by the combination are less than the IDA alone, indicating that chidamide can posttranslationally but not transcriptionally inhibit the IDA-induced expression of the DNA damage repair gene. DNA-damaging agent-inducing DNA lesions could activate specific DNA repair pathways to repair the damage. Thus, highly efficient DDR could promote the survival of cancer cells after exposed to DNA-damaging agents, while DNA repair defects accelerated cancer cell death. At the end of this study, we further explore the consequence of CD34^+^CD38^−^ KG1α cells after DNA damage. We found that chidamide plus IDA eliminates AML stem cell by triggering the activation of caspase-3 and PARP, as indicated by its action on the cleavage of caspase-3 and PARP, but not caspase-8 and caspase-9.

## Conclusions

Despite its limitations, our present study demonstrated that chidamide could sensitize CD34^+^CD38^−^ KG1α, Kasumi cells, and primary refractory or relapsed AML CD34^+^ cells to conventional chemotherapy, and intensified the IDA-induced cell cycle, which was partly through enhancing DNA damage and suppressing the phosphorylation of DNA repair protein. The clinical benefits of adding chidamide into a standard DNA-damaging agent-based chemotherapy regimen should be examined in more AML patients, especially therapy-resistant ones.

## Additional files

Additional file 1: Table S1. The sequences of the primers and the sizes of the amplified fragments. (DOC 17 kb)

Additional file 2: Figure S1. Chidamide synergized IDA-, DNR- or Ara-C-induced apoptosis in both CD34^+^CD38^−^ Kasumi cells. CD34^+^CD38^−^ Kasumi cells were exposed to the indicated concentrations of IDA, DNR, or Ara-C with or without 0.75 μM chidamide for 24, 48, and 72 h (A), with or without 0.5 or 0.75 μM chidamide for 72 h (B), after which flow cytometric analysis was performed to determine the percentage of Annexin V^+^ cells. Horizontal lines represent the mean ± S.D. from three independent experiments. *P < 0.05. (PPT 166 kb)

Additional file 3: Table S2. Summary of CIs generated from the isobologram of increasing concentrations of chidamide and IDA, DNR, or Ara-C for CD34^+^CD38^−^ Kasumi cells. (DOC 19 kb)

Additional file 4: Figure S2. SAHA in combination with IDA, DNR, or Ara-C inhibited CD34^+^CD38^−^ KG1α cell proliferation and induced cell apoptosis. CD34^+^CD38^−^ KG1α cells were exposed to the indicated concentrations of IDA, DNR, or Ara-C with or without 0.6 μM SAHA (IC 30, 0.6 μM) for 24, 48, and 72 h, after which cell viability effect was analyzed by CCK-8 assay (A) or apoptotic ratios were determined by Annexin V staining and flow cytometry(B). Percent of viability was normalized with DMSO-treated control. Values in the figure were expressed as the mean ± S.D. from three independent experiments.*P < 0.05. (PPT 223 kb)

## Abbreviations

AML: Acute myeloid leukemia
Ara-C: Cytarabine
CI: Combination index
DNA: Deoxyribonucleic acid
DNR: Daunorubicin
HDACs: Histone deacetylases
IDA: Idarubicin
LDH: Lactic dehydrogenase
LSC: Leukemia stem cell
WBC: White blood cell
