# Supplementary material for: Cooperative effect of chidamide and chemotherapeutic drugs induce apoptosis by DNA damage accumulation and repair defects in acute myeloid leukemia stem and progenitor cells
Source: Clin Epigenetics. 2017 Aug 14;9:83. doi: 10.1186/s13148-017-0377-8 (PMC5556349; doi:10.1186/s13148-017-0377-8)
Supplement: Supplementary file 1 — The sequences of the primers and the sizes of the amplified fragments. (DOC 17 kb) [file 13148_2017_377_MOESM1_ESM.doc]

Table S1. The sequences of the primers and the sizes of the amplified fragments

| gene | Forward | Reverse | Sizes |
| --- | --- | --- | --- |
| BRCA1 | ACCTTGGAACTGTGAGAACTCT | TCTTGATCTCCCACACTGCAATA | 136 |
| ATM | TTGATCTTGTGCCTTGGCTAC | TATGGTGTACGTTCCCCATGT | 142 |
| CHK1 | ATATGAAGCGTGCCGTAGACT | TGCCTATGTCTGGCTCTATTCTG | 183 |
| CKH2 | TTATCTGCCTTAGTGGGTATCCA | CTGTCGTAAAACGTGCCTTTG | 180 |
| GAPDH | GGAGCGAGATCCCTCCAAAAT | GGCTGTTGTCATACTTCTCATGG | 197 |
