# Supplementary material for: Cooperative effect of chidamide and chemotherapeutic drugs induce apoptosis by DNA damage accumulation and repair defects in acute myeloid leukemia stem and progenitor cells
Source: Clin Epigenetics. 2017 Aug 14;9:83. doi: 10.1186/s13148-017-0377-8 (PMC5556349; doi:10.1186/s13148-017-0377-8)
Supplement: Supplementary file 3 — Summary of CIs generated from the isobologram of increasing concentrations of chidamide and IDA, DNR, or Ara-C for CD34+CD38− Kasumi cells. (DOC 19 kb) [file 13148_2017_377_MOESM3_ESM.doc]

Table S2. Summary of CIs generated from the isobologram of increasing concentrations of chidamide and IDA-, DNR- or Ara-C for CD34^+^CD38^-^ kasumi cells

| Drugs(nM) | FA | Chidamide (µM) | FA*_Chidamide_* | FA*_Comb_* | *CI* |
| --- | --- | --- | --- | --- | --- |
| IDA | FA*_IDA_* |  |  |  |  |
| 20 | 0.143 | 0.5 | 0.031 | 0.220 | 0.736 |
| 20 | 0.143 | 0.75 | 0.039 | 0.274 | 0.619 |
| 40 | 0.354 | 0.5 | 0.031 | 0.475 | 0.746 |
| 40 | 0.354 | 0.75 | 0.039 | 0.609 | 0.544 |
|  |  |  |  |  |  |
| DNR | FA*_DNR_* |  |  |  |  |
| 100 | 0.143 | 0.5 | 0.045 | 0.184 | 0.873 |
| 100 | 0.143 | 0.75 | 0.051 | 0.264 | 0.696 |
| 200 | 0.413 | 0.5 | 0.045 | 0.459 | 0.914 |
| 200 | 0.413 | 0.75 | 0.051 | 0.519 | 0.816 |
|  |  |  |  |  |  |
| Ara-C | FA *_Ara-C_* |  |  |  |  |
| 500 | 0.224 | 0.5 | 0.036 | 0.305 | 0.620 |
| 500 | 0.224 | 0.75 | 0.045 | 0.354 | 0.478 |
| 1000 | 0.344 | 0.5 | 0.036 | 0.460 | 0.560 |
| 1000 | 0.344 | 0.75 | 0.045 | 0.518 | 0.427 |

CI less than 1.0 indicates synergistic effect. FA*_Chidamide_* indicates fraction of cytotoxicity by chidamide alone; FA*_IDA,_* FA*_DNR_* and FA *_Ara-C ,_* fraction of cytotoxicity by IDA, DNR or Ara-C alone; FA*_Comb,_* fraction of cytotoxicity by chidamide plus IDA, DNR or Ara-C.
